# Microvesicles in plasma reflect coronary flow reserve in patients with cardiovascular disease

**DOI:** 10.1152/ajpheart.00869.2020

**Published:** 2021-04-02

**Authors:** Paulina Bryl-Górecka, Kreema James, Kristina Torngren, Inger Haraldsson, Li-Ming Gan, Sara Svedlund, Björn Olde, Thomas Laurell, Elmir Omerovic, David Erlinge

**Affiliations:** ^1^Department of Cardiology, Clinical Sciences, Lund University, Lund, Sweden; ^2^Department of Molecular and Clinical Medicine, Institute of Medicine, Sahlgrenska Academy, University of Gothenburg, Gothenburg, Sweden; ^3^Department of Cardiology, Sahlgrenska University Hospital, Gothenburg, Sweden; ^4^Early Clinical Development, IMED Biotech Unit, AstraZeneca R&D, Gothenburg, Sweden; ^5^Department of Clinical Physiology, Sahlgrenska University Hospital, Gothenburg, Sweden; ^6^Department of Biomedical Engineering, Lund University, Lund, Sweden

**Keywords:** atherosclerosis, biomarkers, coronary flow reserve, microvesicles

## Abstract

High levels of microvesicles (MVs), a type of extracellular vesicles, are detected in several pathological conditions. We investigated the connection between coronary flow reserve (CFR), a prognostic clinical parameter that reflects blood flow in the heart, with levels of MVs and their cargo, from plasma of patients with cardiovascular disease. The PROFLOW study consists of 220 patients with prior myocardial infarction and measured CFR with transthoracic echocardiography. The patients were divided into high and low CFR groups. Plasma MVs were captured with acoustic trapping. Platelet- and endothelial-derived MVs were measured with flow cytometry, and vesicle lysates were analyzed with proteomic panels against cardiovascular biomarkers. Flow cytometry was further applied to identify cellular origin of biomarkers. Our data show a negative correlation between MV concentration and CFR values. Platelet and endothelial MV levels were significantly increased in plasma from the low CFR group. CFR negatively correlates with the levels of several proteomic biomarkers, and the low CFR group exhibited higher concentrations of these proteins in MVs. Focused analysis of one of the MV proteins, B cell activating factor (BAFF), revealed platelet and not leukocyte origin and release upon proinflammatory stimulus. Higher levels of MVs carrying an elevated concentration of proatherogenic proteins circulate in plasma in patients with low CFR, a marker of vascular dysfunction, reduced blood flow, and poor prognosis. Our findings demonstrate a potential clinical value of MVs as biomarkers and possible therapeutic targets against endothelial deterioration.

**NEW & NOTEWORTHY** We investigated how microvesicles (MVs) from patients with cardiovascular diseases are related to coronary flow reserve (CFR), a clinical parameter reflecting blood flow in the heart. Our results show a negative relationship between CFR and levels of platelet and endothelial MVs. The pattern of MV-enriched cardiovascular biomarkers differs between patients with high and low CFR. Our findings suggest a potential clinical value of MVs as biomarkers of reduced blood flow and proatherogenic status, additional to CFR.

## INTRODUCTION

Endothelial dysfunction is a known contributor to atherosclerosis and development of coronary artery disease (CAD). Its major causes are downregulation of nitric oxide (NO) production, release of proinflammatory molecules, and subsequently a decrease in blood flow. Furthermore, endothelial dysfunction is associated with increased platelet aggregation, coagulation, and imbalanced fibrinolysis (1, 2). The transition of endothelial dysfunction into atherosclerosis is a complex process that depends on lipid accumulation, leukocyte adhesion to the vascular wall, and vascular smooth muscle cell proliferation. This in turn leads to oxidative and hemodynamic stress (1, 3). Recent results have pointed to the importance of extracellular vesicles as additional contributors to the development of both endothelial dysfunction and atherosclerosis (4, 5).

Extracellular vesicles (EVs) are an overall designation for several subtypes of vesicles, such as apoptotic bodies, microvesicles (MVs), and exosomes (4), where the subtype division is based on size or biogenesis. MVs are membrane-derived vesicles, usually between 30 and 1,000 nm, that are formed by plasma membrane shedding. They carry biologically active receptors and phospholipids on their surface, as well as a molecular cargo in their lumen. The vast majority of MVs derive from cellular components of blood, such as platelets, erythrocytes, and leukocytes (4, 5). Another important source of plasma MVs is endothelial cells, which, upon activation (6), produce MVs presenting E-selectin (CD62E).

Several studies have indicated a strong connection between MVs and pathological processes (7). Platelet and endothelial MVs have been described as the hallmarks of a range of diseases related to the cardiovascular system, such as myocardial infarction (MI), CAD, and hypertension (4, 5, 8). It was shown that MVs generated from activated endothelial cells enhance platelet aggregation ex vivo through a von Willebrand factor (vWF)-dependent mechanism of crosslinking (9) and contribute to the development of atherosclerosis through, e.g., enhancing clotting cascade, interacting with monocytes, and facilitating EC proliferation (10). They can also modulate vascular smooth muscle cell differentiation and thus plaque progression (11). Previous studies have shown that plasma MVs carry many different classes of molecules, such as proteins (12–15), miRNAs, and long noncoding RNAs (16) that could be of mechanistic and diagnostic interest. Our group has already demonstrated a biomarker potential of MVs isolated with acoustic trapping and analyzed with mass spectrometry (17) flow cytometry and Olink proximity extension assays (PEA) (18, 19).

One widely used clinical parameter for assessing vascular tone and vascular blood flow is coronary flow reserve (CFR). CFR captures both flow-limiting atherosclerotic lesions in the epicardial segments of the coronary arteries as well as microvascular dysfunction and is a strong predictor of cardiovascular events (20–23). Subjects with high CFR values usually have better coronary circulation, and their cardiovascular status is superior to patients with low CFR, as CFR is directly related to oxygen and nutrients delivery to the heart during higher demand (21, 24).

Here, we present a MV characterization of 220 patients with measured CFR by transthoracic echocardiography in the PROFLOW (PROspective Evaluation of Coronary FLOW Reserve and Molecular Biomarkers in Patients with Established Coronary Artery Disease) study (25). We investigated if there is a connection between CFR and MVs. Platelet and endothelial MVs, isolated with a novel vesicle enrichment method, acoustic trapping, were counted with flow cytometry. Furthermore, EV lysates were analyzed with a multiplex proximity extension assay to measure potential biomarkers in MVs.

## MATERIALS AND METHODS

### Study Design

The PROFLOW prospective, exploratory, and open study has previously been described by Haraldsson et al. (25). It consists of 619 high-risk patients with verified CAD that were recruited by the Department of Cardiology at Skåne University Hospital in Lund and Sahlgrenska University Hospital in Gothenburg, Sweden. Sample size, power calculations, and numbers of subjects at each stage of study were described previously (25). Here, we present the data from 220 patients included in Lund. The patients with high risk for future major adverse cardiovascular events were recruited based on the data from Swedish Coronary Angiography and Angioplasty Registry (SCAAR). Eligibility criteria included prior MI (>3 mo, <5 yr) and are presented in Supplemental Table S1 (all Supplemental material is available at https://doi.org/10.6084/m9.figshare.13415033). CFR measurements and sample collection were done between July 12, 2013, and December 16, 2015. All procedures were performed after obtaining an informed written consent and in agreement with Declaration of Helsinki. The study was approved by the ethics committee in Gothenburg.

### Sample Collection

Blood from fasting patients was collected into EDTA tubes and centrifuged within 30 min. Obtained cell-free plasma was aliquoted and frozen in −80°C.

### CFR Assessment

CFR was measured in LAD as described previously (25) with Sequoia C256 ultrasound system (Acuson Siemens, Mountain View, USA). The mid to distal part of LAD was identified using 3.5 MHz color transthoracic Doppler in a modified 2/3-chamber view. Flow velocity signals at rest and during peripheral intravenous adenosine infusion (140 μg/min/kg) over 5 min were recorded with pulsed wave Doppler. Mean diastolic flow velocity at baseline and during peak hyperemia was measured by tracing of the diastolic Doppler flow signals. CFR was calculated as the ratio between the hyperemic and baseline flow velocity values. All evaluation CFR was performed by one experienced sonographer at the core laboratory (Sahlgrenska University Hospital) blinded to the information about patients’ characteristics.

### MV Preparation with Acoustic Trapping Platform

The acoustic trapping platform, a novel method for enrichment of MVs from patient plasma samples, has been previously described by our group for flow cytometric and proteomic analysis of vesicles (17–19, 26). MVs were trapped from frozen plasma in an automated setup consisting of acoustic trapping unit and a robotic 96-well plate (AcouTrap, AcouSort AB, Lund, Sweden). For flow cytometric analysis, samples were thawed at 37°C and 50 μL of 1:4 diluted plasma was aspirated at 25 μL/min into the already trapped seed particle (12-µm polystyrene beads, Fluka) cluster. Trapped MVs were then washed and released in 100 μL of DPBS. For multiplex PEA, 200 μL of 1:4 diluted plasma sample, corresponding to 50 μL of undiluted plasma, was aspirated at 25 μL/min across the seed particle cluster and released in 50 μL of DPBS. To monitor trapping efficiency, pooled plasma from healthy volunteers served as internal control. Acoustic trapping efficiency was calculated based on concentration of CD42a+ MVs and remained at a stable and high level throughout the whole experiment.

### Flow Cytometry of Acoustically Trapped MVs

Flow cytometry was performed as previously described (18). Eluted MV suspension (100 μL) was stained with 3 μL of PE-conjugated antibodies (Abs) against endothelial activation marker E-selectin (CD62E) or platelet marker glycoprotein IX, known as CD42a (BD Biosciences). Samples were analyzed with an Accuri C6 cytometer (BD Biosciences).

### Multiplex Proximity Extension Assay of Patient Samples

Olink multiplex PEA and sample preparation for this procedure were performed, as previously described, on total EV fraction (18). Protein concentration was adjusted to 0.5 μg/μL for all samples. Two Olink proteomic panels were used: CVD II and CVD III. The proteomic data were analyzed based on normalized protein expression (NPX) values of detected proteins (27).

### Cellular Origin of Circulating MV-Bound BAFF in Plasma

To determine cellular origin of circulating B cell activating factor (BAFF) + MVs, flow cytometric analysis was performed on selected PROFLOW cohort samples with an Apogee A60 cytometer (Apogee Flow Systems, Hemel Hempstead, UK). Size-calibrated silica beads (ApogeeMix, Apogee Flow Systems) were used to establish MV gate (Supplemental Fig. S1). Fluorescence gates were created based on Ab isotype controls: PE mouse IgG1κ, FITC mouse IgG1κ, and Alexa Fluor 488 mouse IgG1κ (Supplemental Fig. S2), as well as Abs in DBPS. All Abs were centrifuged 20,000 *g* for 30 min in 4°C prior application, to remove Ab aggregates. Patient plasma was diluted 1:200 with DPBS, and 200 μL was stained for 30 min in RT with 2 μL of Abs against cellular origin markers and BAFF and directly analyzed. The gating strategy is presented in Supplemental Fig. S3A. CD42b PE and FITC clone HIP1, CD31 PE clone WM59, CD45 FITC clone HI30, CD16 PE and FITC clone 3G8, CD62E PE clone 68-5H11, CD36 PE clone CB38, and CD144 FITC clone 55-7H1 were purchased from BD Biosciences. CD235a FITC clone KC16 was purchased from Beckman Coulter. BAFF Alexa Fluor 488 was purchased from Bio-Techne. BAFF PE clone 1D6 was purchased from Thermo Fisher Scientific.

### BAFF Presence on Megakaryocyte Cell Line-Derived MVs

MEG01 cells were cultured in T25 flasks in RPMI medium (Thermo Fisher Scientific) supplemented with nonessential amino acids and Na-pyruvate. 10 ng/μL of TNFα was added to the medium. After overnight incubation, 10 mL of medium was collected, centrifuged 300 *g* and 2,500 *g* for 10 min each to remove cellular debris, and ultracentrifuged at 100,000 *g* for 1 h in 4°C. The remaining vesicle-free supernatant was discarded, and the MV-rich pellet was suspended in 500 μL of DPBS. For analysis with Apogee cytometer, MV suspension was diluted 50× in DPBS and 200 μL was stained with 2 μL of BAFF PE Ab for 30 min. The flow cytometric analysis was performed as described in *Cellular Origin of Circulating MV-Bound BAFF in Plasma*. The gating strategy is shown in Supplemental Figs. 1, 2, and 3*B*.

### Effect of MVs from High and Low CFR Groups on ICAM-1 Expression in ECs

Human coronary artery endothelial cells were cultured in six-well plates in Human MesoEndo Endothelial Cell Medium (Cell Applications). Sixty-five microliters of six plasma samples chosen from high and low CFR groups were pooled separately (390 μL total for each group) and centrifuged 20,000 *g* for 45 min in RT to obtain MV pellet. The pellet was reconstituted in 500 μL of DPBS, and 250 μL of MV suspension was added to the semiconfluent cells. After an overnight incubation, ECs were gently harvested, washed, and resuspended in 500 μL DPBS. Cell suspension (50 μL) was further diluted with 450 μL of DPBS and stained with 10 μL of CD54/intercellular adhesion molecule 1 (ICAM-1) PE Ab for 25 min in 4°C. For FCM analysis with Apogee cytometer, the cell suspension was washed and resuspended in 500 μL of PBS. The fluorescence-based gates were based on isotype controls and unstained samples.

### Statistics

Patients were divided into low or high CFR group based on the CFR median (2.96) due to relatively high CFR values in the cohort and to create equal groups. The results of Shapiro test revealed that the flow cytometric and proteomic data did not exhibit normal distribution and a nonparametric approach was applied. Mann–Whitney *U* test for unpaired data was used for comparisons of MVs and proteins in low and high CFR groups. One-way ANOVA was used to explore the effect of circulating MVs from high and low CFR groups on ECs. *P* values <0.05 were considered as statistically significant. Spearman’s coefficients were calculated to determine correlations, and simple linear regression was used to study the relationship between MVs and proteins. The relationship between CFR and certain factors was measured with multiple linear regression. The adjustment variables were sex, body mass index (BMI), smoking, age, diabetes, hypertension, dyslipidemia, LDL levels, as well as platelet and endothelial MV concentration. We identified and removed one outlier (CFR = 6.4) to satisfy the normality of the residual assumption. The analysis of Olink proteomic panels was done on NPX (normalized protein expression) values. Out of 180 biomarkers present in Olink CVD II and III panels, 68 were detected in more than 50% patients. Proteins with 50% ≤ missing data (NPX values lower than limit of detection) were removed from the analysis. Analyses were performed with GraphPad Prism software version 7.0a (GraphPad Software Inc., La Jolla, CA).

## RESULTS

### Study Design and Patient Information

The PROFLOW study is a clinical investigation where CFR values, as well as demographic and biochemical parameters, were measured in patients with previous MI (25). The CFR parameter is calculated on the maximal increase in arterial blood flow during infusion of adenosine in relation to resting blood flow. It is used to detect both flow-limiting plaques in coronary arteries and microvascular dysfunction (28). CFR measurement with transthoracic echocardiography is an established technique that carries strong prognostic information and is an alternative to invasive methods (21).

The study group consists of 220 subjects with a mean age of 67. Detailed patient data are presented in Table 1. The subjects were included in the study after providing an informed written consent. The numbers of subjects at each stage of study were described previously (25). Compared with data published elsewhere (25), this study focuses on connection between CFR and EVs. Factors associated with low CFR included age, heart rate, smoking, and dyslipidemia (25). We were able to measure EVs in 220 patients from our site.

**Table 1.** PROFLOW study patient information

Parameters	All Patients	High CFR	Low CFR
*n*	220	108	112
CFR, means ± SD	2.96	3.41*	2.45
Age, yr	67 ± 6.3	67 ± 6.4	67 ± 6.1
Height, cm	174 ± 8.7	174.5 ± 8.4	172.9 ± 8.8
Weight, kg	83 ± 13.3	83.4 ± 13.4	82.5 ± 13.2
Men, *n* (%)	183 (83)	93 (86)	90 (80)
Women, *n* (%)	37 (17)	15 (14)	22 (20)
Hypertension, *n* (%)	124 (56)	61 (56)	63 (56)
Diabetes, *n* (%)	53 (24)	23 (22)	30 (27)
Dyslipidemia, *n* (%)	99 (61)	49 (45)	50 (45)
Angina pectoris, *n* (%)	39 (17)	15 (14)	24 (21)
NSTEMI, *n* (%)	198 (89)	94 (86)	104 (92)
STEMI, *n* (%)	24 (11)	15 (14)	9 (8)
Stenosis†, *n* (%)	13 (6)	5 (5)	8 (7)
Presence of plaque, *n*s‡ (%)	162 (74)	74 (68)	88 (78)
Current smokers, *n* (%)	20 (9)	7 (6)	13 (11)
Previous smokers, *n* (%)	125 (57)	61 (56)	63 (56)
Low-density lipoprotein, mmol/L	2.1 ± 0.8	2.1 ± 0.8	2.1 ± 0.8
Triglycerides, mmol/L	1.1 ± 0.6	1.1 ± 0.6	1.1 ± 0.6
C-reactive protein, mg/L, means ± SD	1.7 ± 2.0	1.6 ± 2.0	1.7 ± 2.1
Neutrophils, x10^3^/mm^3^, means ± SD	4.0 ± 1.2	3.9 ± 1.1	4.0 ± 1.3
GFR, mL/min/1.73 m^3^, means ± SD	85.5 ± 24.0	85.9 ± 22.4	85.1 ± 25.3
Medication			
Statins, *n* (%)	201 (91)	98 (91)	102 (91)
Acetylsalicylic acid, *n* (%)	212 (96)	105 (97)	108 (96)
B-blocker, *n* (%)	174 (79)	84 (78)	90 (80)
Diuretics, *n* (%)	40 (18)	21 (19)	19 (18)
P2Y12 receptor antagonists, *n* (%)	60 (27)	31 (29)	30 (27)
ACEi, *n* (%)	126 (57)	58 (53)	68 (61)
ARB, *n* (%)	59 (27)	28 (26)	31 (28)
CCB, *n* (%)	34 (15)	20 (18)	14 (12)

Values are means ± SD or *n* (%); *n*, number of participants. ACEi, angiotensin-converting enzyme inhibitors; ARB, angiotensin receptor blockers; CCB, calcium channel blockers; CFR, coronary flow reserve; GFR, glomerular filtration rate; NSTEMI, non-ST elevation myocardial infarction; STEMI, ST elevation myocardial infarction.

* Statistically significant, *P* < 0.05, Mann–Whitney *U* test.

† Defined as a 50% lumen narrowing.

‡ Evaluated in short-axis view of the carotid bifurcation.

### Relationship between CFR and MVs

**Figure 1.**
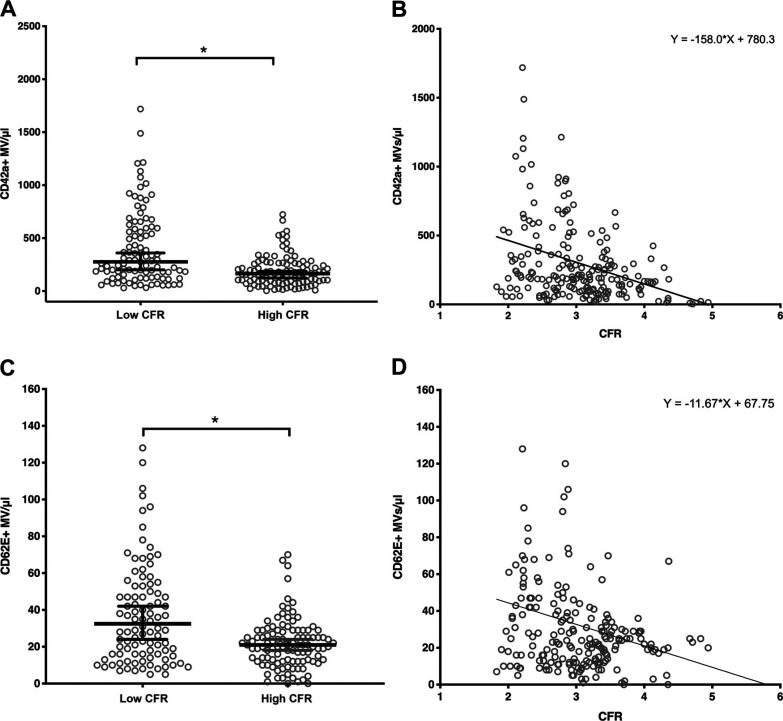
CFR negatively correlates with levels of platelet and endothelial MVs. *A* and *C*: scatter plots showing median ± 95% CI. Patients with high CFR have significantly lower concentration of platelet (*A*) and endothelial (*C*) MVs in plasma, compared with the low CFR group. Mann–Whitney *U* test for unpaired data. CD42a+ and CD62E+ MVs: *n* = 100 (low CFR group), *n* = 107 (high CFR group). *Statistically significant, *P* < 0.0001 for both platelet and endothelial MVs. *B* and *D*: CFR and MV correlation plots. Spearman’s rho for platelet MVs = −0.34, *n* = 207. Spearman’s rho for endothelial MVs = −0.24, *n* = 207. *P* < 0.0001 and *P* = 0.001 for platelet and endothelial MVs, respectively. The curves demonstrate the results of simple linear regression. CFR, coronary flow reserve; MVs, microvesicles.

As platelet and endothelial MVs contribute to endothelial dysfunction and development of atherosclerosis, we decided to investigate their relationship with CFR values. Both CD42a+ platelet MVs and CD62E+ endothelial MVs were counted, and their concentrations were compared with high and low CFR groups, defined by the median, as to create groups with equal numbers of patients. We found that the low CFR group had significantly higher concentration of platelet MVs in plasma (median: 276 CD42a+ MVs/μL), compared with the high CFR group (median: 165 CD42a+ MVs/μL) (Fig. 1*A*). The low CFR group was also characterized by more scattered data than the high CFR group. There was a negative correlation (Spearman’s rho = −0.34) between CFR and platelet MV levels (Fig. 1*B*).

**Figure 2.**
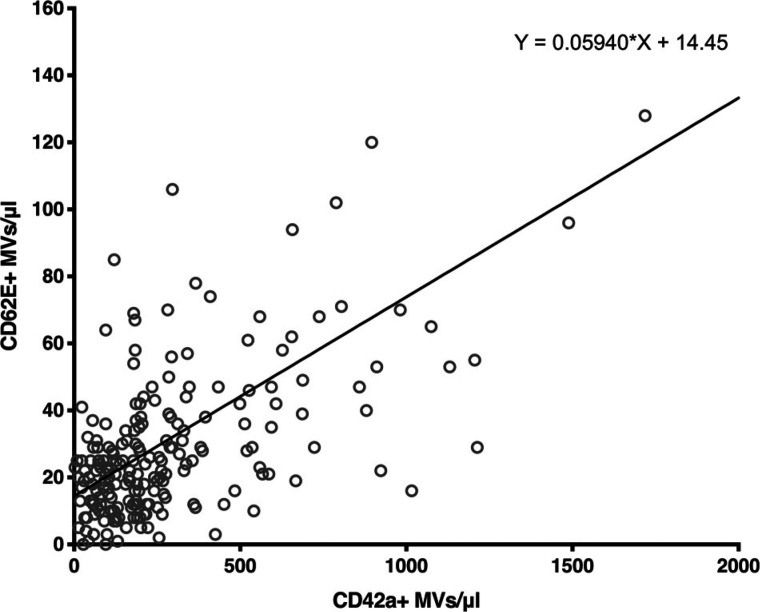
Endothelial MVs positively correlate with platelet MV levels in plasma. Scatter plot. Spearman’s rho = 0.52, *P* < 0.0001; *n* = 207 for both CD42a+ and CD62E+ MVs. MVs, microvesicles.

We also investigated the relationship between CD62E+ endothelial MVs and CFR values. Similarly, the low CFR group was characterized by significantly higher concentration of endothelial MVs in plasma (median: 32.5 CD62E+ MVs/μL), compared with the high CFR group (median: 21 CD62E+ MVs/μL) (Fig. 1*C*) and a more scattered distribution. The correlation between CFR and endothelial MV levels was also negative (Spearman’s rho = −0.24) (Fig. 1*D*). The relationship between platelet and endothelial MV concentrations in plasma was also measured. As shown in Fig. 2, the correlation between CD42a+ and CD62E+ MV levels was found to be positive (Spearman’s rho = 0.52). We did not find any relationship between platelet or endothelial MVs and risk factors (Supplemental Fig. S4), as well as disease and inflammatory burden (Supplemental Fig. S5 and S6).

We also applied explanatory multiple linear regression to explore potential relationship of CFR with levels of platelet and endothelial MVs. The other standard clinical variables included in the model were sex, BMI, smoking, age, diabetes, hypertension, dyslipidemia, and LDL levels. The analysis revealed statistical significance for platelet MV concentration parameter estimate (−0.0006, CI: −0.0009 to −0.0002).

### Proteomic Analysis of Plasma EVs

**Figure 3.**
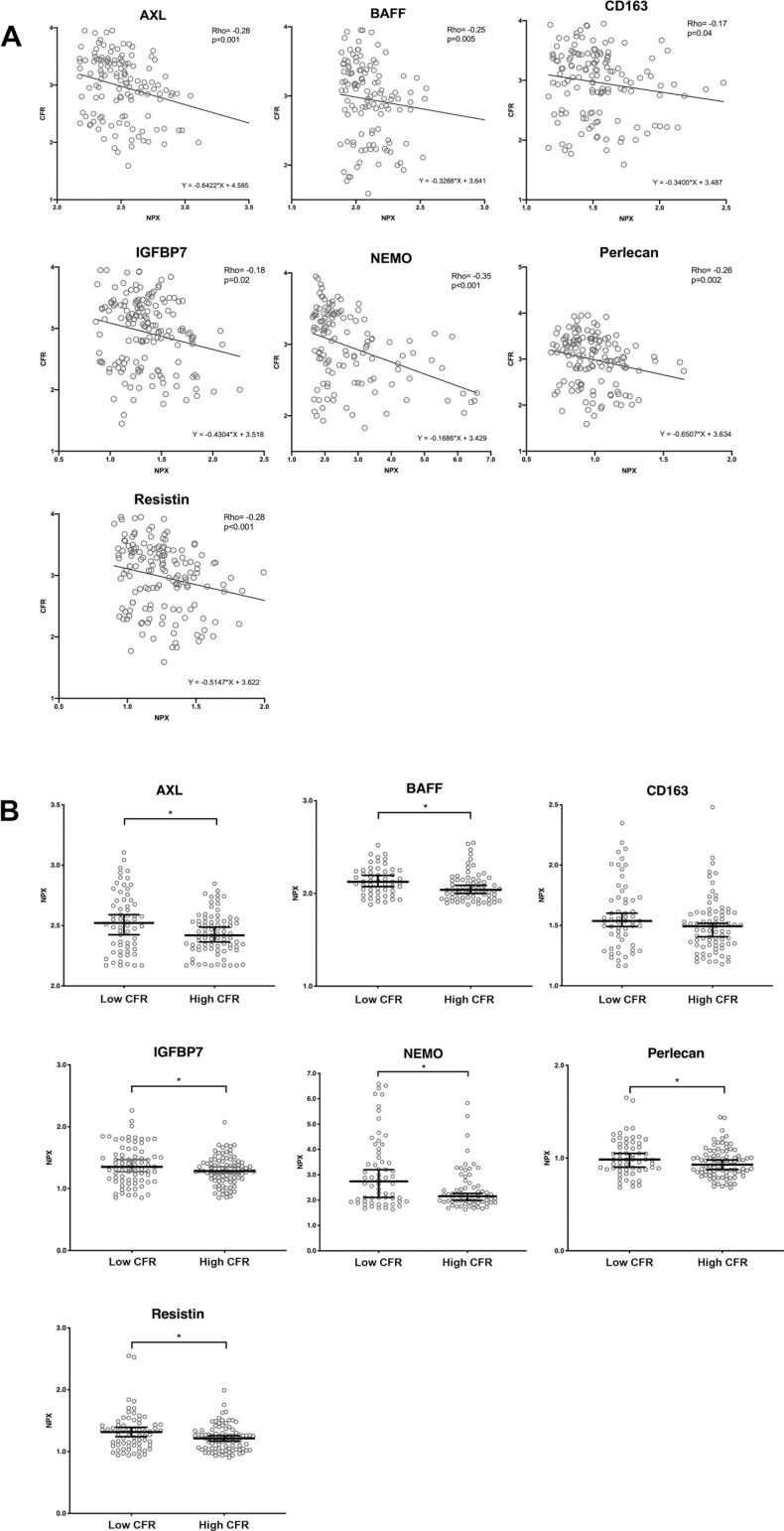
CFR negatively correlates with seven cardiovascular disease-related proteins (*A*) that are lower in MVs from patients with high CFR (*B*). Olink proteomic CVD II and III panels were used to measure 180 cardiovascular biomarkers. *A*: correlation plots showing normalized protein expression (NPX) values of the significantly correlated proteins (*x*-axis), with corresponding CFR (*y*-axis), *P* < 0.05. AXL: *n* = 143, BAFF: *n* = 122, CD163: *n* = 137, IGFBP7: *n* = 166, NEMO: *n* = 130, perlecan: *n* = 145, resistin: *n* = 157. *B*: patients from the high CFR group have significantly lower biomarker concentration (NPX values) in plasma MVs than the low CFR group. Scatter plots presenting median ± 95% CI. Mann–Whitney *U* test for unpaired data. *Statistically significant, *P* < 0.05. AXL: *n* = 62 (low CFR), 81 (high CFR); BAFF: *n* = 52 (low CFR), 70 (high CFR); CD163: *n* = 58 (low CFR), 79 (high CFR); IGFBP7: *n* = 75 (low CFR), 91 (high CFR); NEMO: *n* = 62 (low CFR), 68 (high CFR); perlecan: *n* = 59 (low CFR), 86 (high CFR); resistin: *n* = 68 (low CFR), 89 (high CFR). BAFF, B cell activating factor; CFR, coronary flow reserve; IGFBP7, insulin-like growth factor-binding protein 7; MVs, microvesicles; NEMO, NF-κB essential modulator.

As the results of the previous sections indicated that the release of MVs is connected to CFR, we next analyzed if this is also reflected in the vesicle content. A proteomic analysis was performed using Olink CVD II and III panels where 180 potential cardiovascular biomarkers were measured in EVs isolated from patient plasma. The biomarker measurements were adjusted to the protein content in the EV lysates and not based on the total plasma protein level. A complete list of proteins included in the panels is presented in Supplemental Table S2. We identified 67 proteins present in the EV lysates. Statistical analysis of CFR and NPX levels revealed seven negatively correlating proteins (Fig. 3*A*): NF-κB essential modulator (NEMO or IKBKG) (rho = −0.35), resistin (RETN) (rho = −0.28), tyrosine-protein kinase receptor UFO (AXL) (rho = −0.29), perlecan (PLC or HSPG2) (rho = −0.26), B cell activating factor or BAFF (TNSF13B) (rho = −0.25), insulin-like growth factor-binding protein 7 (IGFBP7) (rho = −0.18), and CD163 (rho = −0.17). We did not find any positively correlating proteins with CFR. Complete list of detected proteins and corresponding Spearman’s rho values is included in Supplemental Table S3.

After comparing high and low CFR groups, we observed that the high CFR group exhibited lower concentration of all aforementioned proteins in EVs, excluding CD163 (Fig. 3*A*). This marker did not reach statistical significance, although there is a visible trend toward lower values in the high CFR group. A complete list of calculated median values for low and high CFR groups is included in Supplemental Table S4.

**Figure 4.**
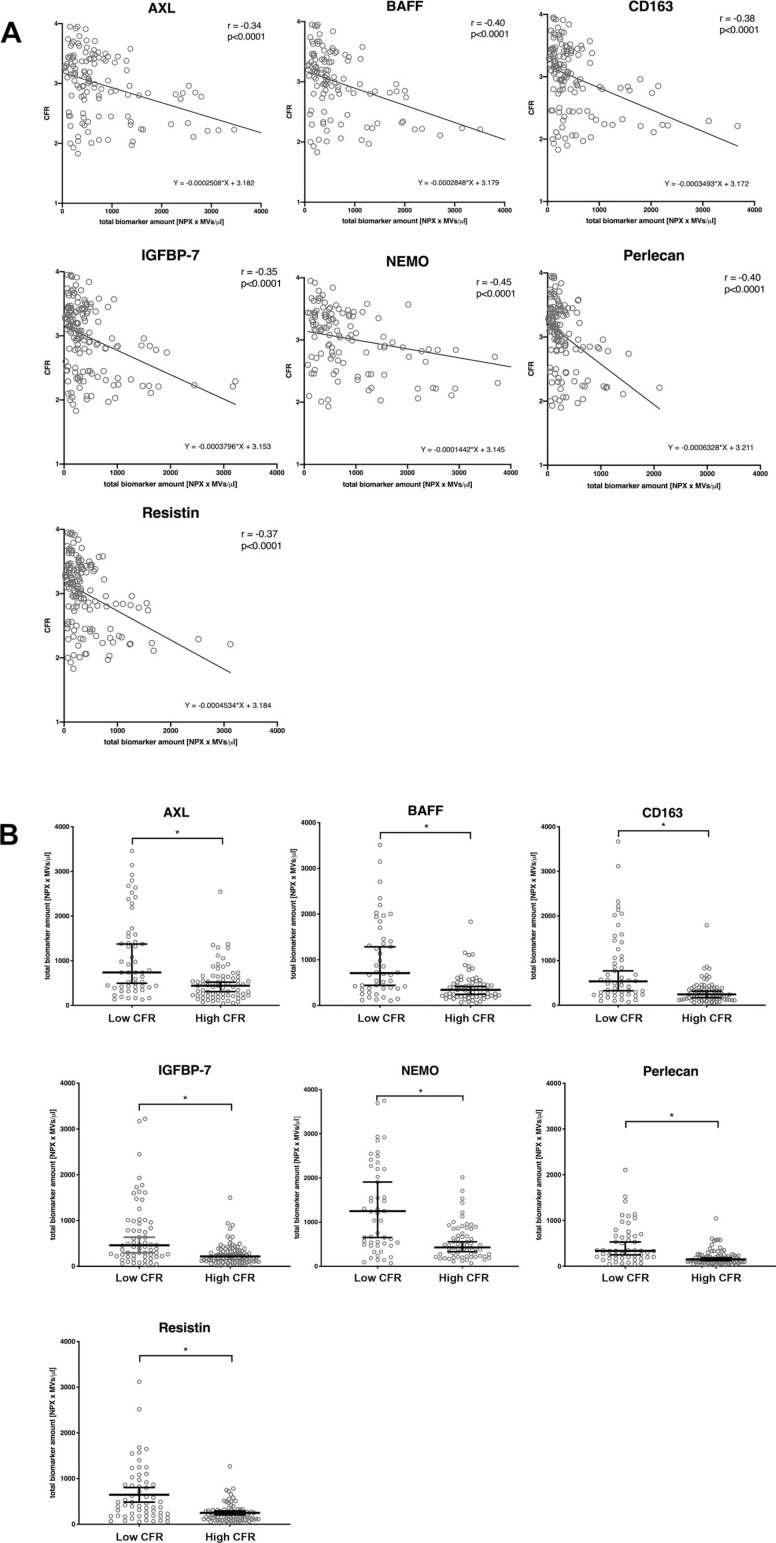
CFR negatively correlates to a parameter based on platelet MV concentration and protein NPX (*A*), and patients with high CFR have lower levels of this parameter. *A*: correlation plots showing normalized protein expression (NPX) values of the significantly correlated proteins, multiplied by platelet MV concentration (*x*-axis), with corresponding CFR (*y*-axis), *P* < 0.0001. AXL: *n* = 129, BAFF: *n* = 115, CD163: *n* = 132, IGFBP7: *n* = 151, NEMO: *n* = 121, perlecan: *n* = 133, resistin: *n* = 145. *B*: scatter plots showing median ± 95% CI. Mann–Whitney *U* test for unpaired data, *P < 0.05, statistically significant. AXL: *n* = 129, BAFF: *n* = 115, CD163: *n* = 132, IGFBP7: *n* = 151, NEMO: *n* = 121, perlecan: *n* = 133, resistin: *n* = 145. BAFF, B cell activating factor; CFR, coronary flow reserve; MV, microvesicle; IGFBP7, insulin-like growth factor-binding protein 7; MV, microvesicle; NEMO, NF-κB essential modulator.

As the majority of plasma vesicles is derived from platelets, we further measured the relationship between CFR and a parameter calculated based on platelet MV concentration and protein NPX values, representing total biomarker amount. The results revealed even stronger, significant negative correlations for all abovementioned proteins (Fig. 4*A*). Comparison of the parameters between the groups showed that the high CFR group was characterized with significantly lower values, including CD163 (Fig. 4*B*).

### Cellular Origin of Circulating MV-Bound BAFF in Plasma

**Figure 5.**
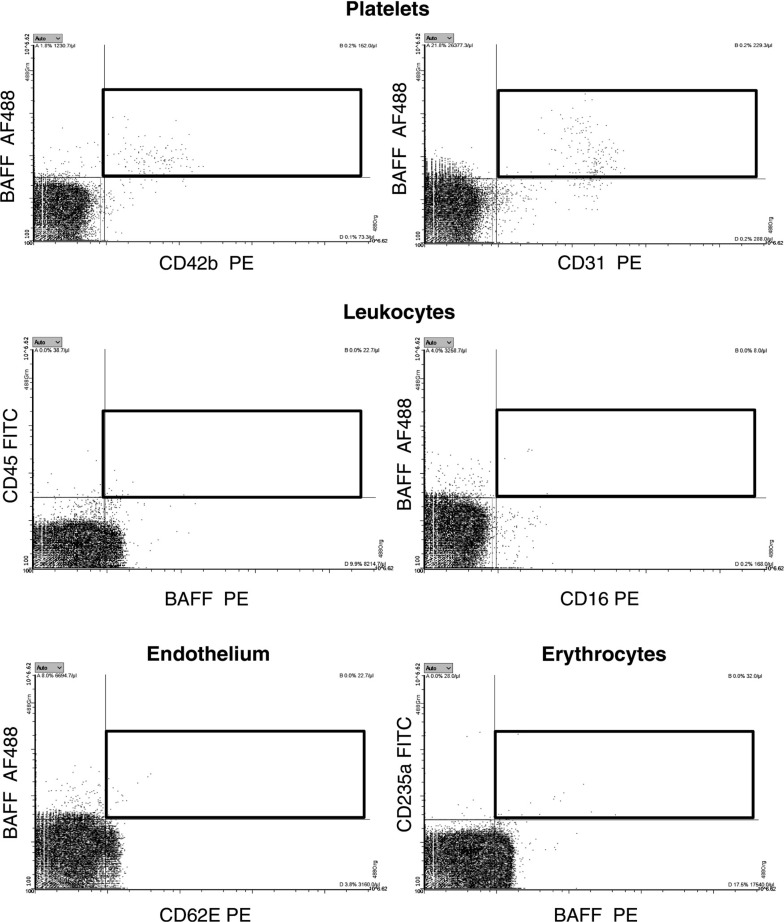
Tracking the origin of circulating BAFF+ MVs in plasma of patients with cardiovascular disease reveals its platelet origin. Platelet-free plasma was costained with BAFF and markers of platelets (*top*), leukocytes (*middle*), endothelial cells (*bottom, left*), and erythrocytes (*bottom, right*). There was a substantial positive costaining of BAFF with CD42b and CD31 platelet markers. Lack or very low costaining was observed for leukocytes, endothelial cells, or erythrocytes. This suggests that BAFF+ MVs in plasma represent a fraction of platelet MVs. BAFF, B cell activating factor; MVs, microvesicles.

**Figure 6.**
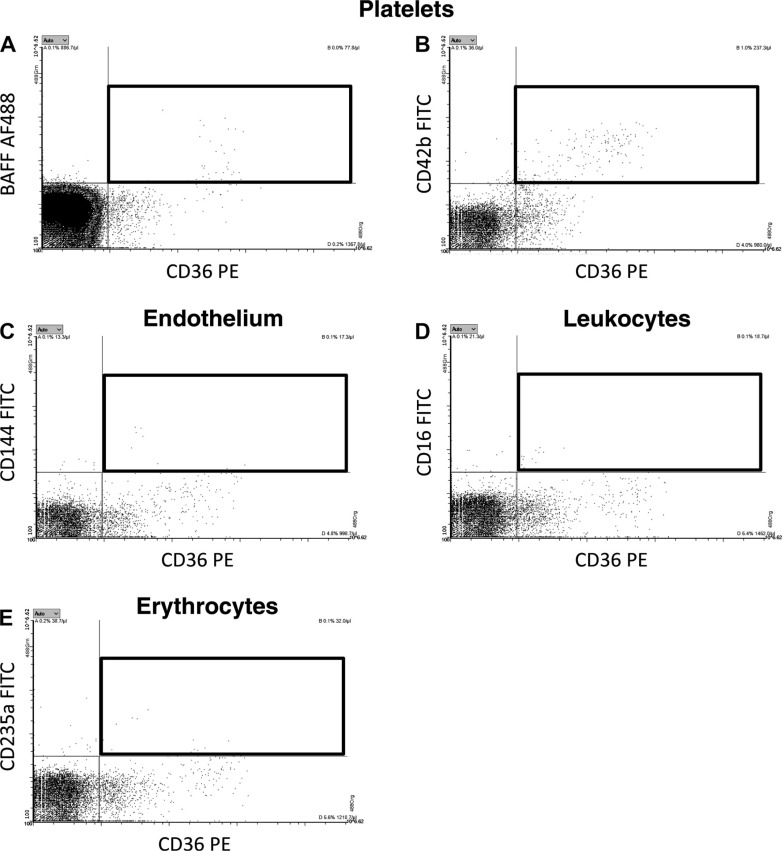
Additional staining performed to track BAFF and CD36 origin in plasma. Platelet-free plasma from patients with cardiovascular disease was costained with BAFF and CD36, an oxidized LDL receptor, present on different types of cells. There was a positive costaining for a substantial fraction of MVs (*A*). Establishing cellular origin of CD36 revealed that platelets (*B*) and not endothelial cells (*C*), leukocytes (*D*), or erythrocytes (*E*) were major sources of CD36. Thus, BAFF+ and CD36+ MVs in plasma represent a fraction of platelet MVs. BAFF, B cell activating factor; MVs, microvesicles.

**Figure 7.**
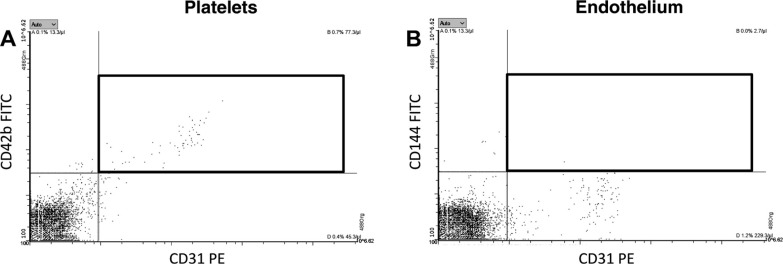
Additional staining performed to track CD31 origin in plasma, as it has been reported to be present on both platelets and endothelial cells. Platelet-free plasma from patients with cardiovascular was costained with CD31 and CD42b, a platelet MV marker (*A*) or CD144, an endothelial marker (*B*). The staining revealed that majority of CD31+ MVs came from platelets (*A*) and not endothelial cells (*B*), thus confirming our data that BAFF+ MVs represent a fraction of platelet MVs. BAFF, B cell activating factor; MVs, microvesicles.

BAFF is a protein that is notable both for being involved in CAD (29) and also for being a novel pharmacological target for anti-inflammatory therapeutics (30). Therefore, we decided to focus on BAFF and identify the cellular origin of circulating BAFF-expressing MVs. We performed FCM analysis of BAFF+ MVs, costained against platelet, leukocyte, and endothelial and erythrocyte markers (Fig. 5). Our data revealed that BAFF-expressing MVs have mainly platelet origin (CD42b and CD31 markers). As CD31 can also be present on ECs, we performed a costaining of CD31 with CD42b and CD144 (endothelial marker) (Fig. 6). It confirmed that BAFF+ CD31+ MVs have platelet (Fig. 6*A*) and not endothelial origin (Fig. 6*B*). Neither leukocytes (CD45 and CD16 markers) nor activated endothelial cells (CD62E marker) or erythrocytes (CD235a marker) were the source of BAFF+ MVs (Fig. 6). We also performed additional costaining of BAFF and CD36, an oxidized LDL receptor present on different types of cells that is involved in proatherosclerotic processes (31) (Fig. 7). It demonstrated that there is a subpopulation of BAFF+ MVs that express CD36 on the surface (Fig. 7*A*). We further identified platelets (Fig. 7*B*) and not ECs, leukocytes, or erythrocytes (Fig. 7, *C*–*E*), as cellular sources of circulating CD36+ MVs in our cohort. Thus, we conclude that BAFF+ CD36+ MVs in plasma represent a fraction of platelet MVs, and generally, BAFF+ MVs are of platelet origin.

### Inflammatory Conditions Stimulate Release of BAFF+ MVs

**Figure 8.**
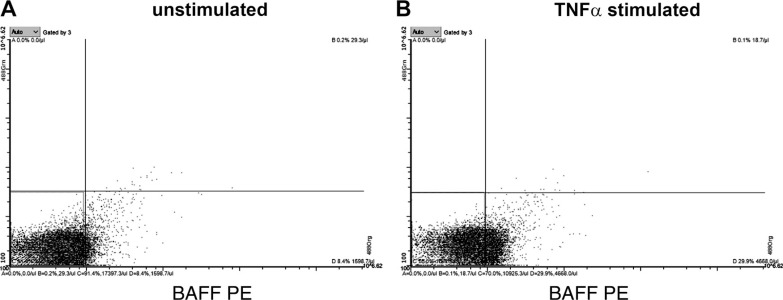
Release of BAFF+ MVs from MEG01 cells is increased upon TNFα stimulation. Megakaryocyte cell line was stimulated with pro-inflammatory conditions to measure release of BAFF+ MVs. The levels of BAFF-expressing MVs increased substantially (*B*) after applying TNFα, compared with the unstimulated MEG01 cells (*A*), pointing at the role of proatherosclerotic conditions in the formation of circulating BAFF+ MVs. BAFF, B cell activating factor; MVs, microvesicles.

To assess if proinflammatory conditions increase a release of BAFF+ platelet MVs, TNFα was used as a stimulant on a megakaryocyte cell line. The FCM analysis revealed that levels of BAFF-expressing MVs increased after TNFα stimulation (Fig. 8*B*), compared with the unstimulated MEG01 cells (Fig. 8*A*). These findings confirm that the release of BAFF+ MVs occurs during proatherosclerotic conditions and explains why its levels are higher in the low CFR group.

### Effect of MVs from High and Low CFR Groups on ICAM-1 Expression in ECs

**Figure 9.**
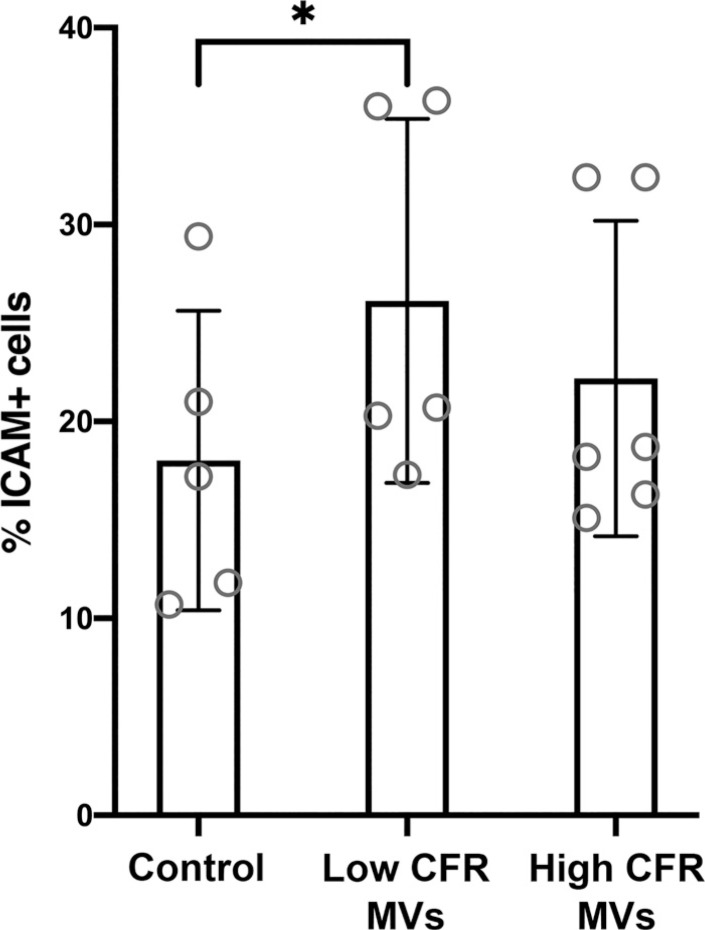
Microvesicles isolated from plasma of the low CFR group significantly increase ICAM-1 expression on endothelial cells. Bar graph showing means ± SD. One-way ANOVA, *n* = 3 experiments with replicates. *Statistically significant, *P* < 0.05. CFR, coronary flow reserve; ICAM-1, intercellular adhesion molecule 1.

To functionally assess the proatherosclerotic impact of circulating MVs on the vasculature, ECs were treated with MVs isolated from selected plasma samples from patients with the highest and the lowest CFR. ICAM-1 presence on the cells was further measured with FCM (Supplemental Fig. S7). As seen in Fig. 9, ECs incubated with MVs from the low CFR group expressed significantly higher levels of ICAM-1 (mean: 26% ICAM-1+ cells) compared with the control cells (mean: 18% ICAM-1+ cells). Incubation of ECs with MVs from the high CFR group mildly increased ICAM-1 expression (mean: 22% ICAM-1+ cells) compared with the control cells, however without reaching a statistical significance.

## DISCUSSION

Endothelial dysfunction and subsequent atherosclerosis are major causes of CAD and result in reduced CFR values (21). Although it is well known that both platelet and endothelial MVs take part in these processes, the relation between MVs and CFR has not been investigated until now. In our study, CFR of 220 patients with CAD was measured and MVs were isolated from the corresponding plasma samples. The outcome displays a significant difference in the pattern of MV-associated cardiovascular biomarkers, between patients with high and low CFR.

**Figure 10.**
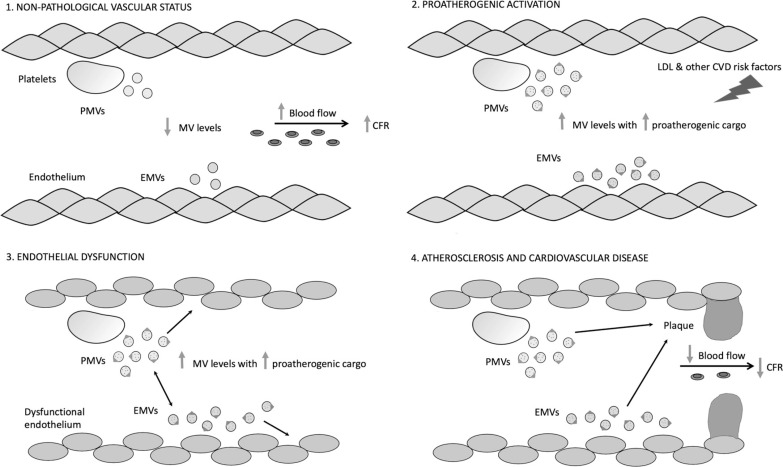
Proposed MV connection with decreased CFR. In physiological conditions, platelets and endothelial cells release low levels of platelet MVs (PMVs) and endothelial MVs (EMVs) (1). CVD risk factors, e.g., high LDL, contribute to platelet and endothelial activation (2) and a substantial increase in MV production, carrying an increased load of procoagulant and proinflammatory cargo. This proatherogenic status in the vessel leads to endothelial dysfunction (3) and atherosclerotic plaque progression (4) that further contribute to a decrease in the blood flow, finally manifested as low CFR, as well as a development of CVD. CFR, coronary flow reserve; MV, microvesicle.

The results show a higher concentration of both platelet and endothelial MVs in the plasma of the low CFR group, and both correlated negatively with CFR. The statistical analysis also revealed that for each increase in 100 platelet MVs/μL, the CFR value decreases on average by 0.06 (CI: -0.08 to -0.02). It is possible that proinflammatory and proatherogenic MVs contribute to reduced CFR. We believe that a high concentration of platelet MVs in plasma negatively affects vascular physiology, mainly through contributing to atherosclerotic plaque formation (4, 29, 32) and by stimulation of platelet thromboxane A2 production (33). MVs generated by a dysfunctional endothelium are known to negatively affect healthy endothelial cells by decreasing NO production and vascular relaxation (34), thus amplifying the dysfunction and contributing to lower CFR values. MVs released by aging ECs lead to calcification in the vessel and further deterioration of the plaque (33). Based on the current knowledge, we propose a model of endothelial MV influence on blood vessels, where high numbers negatively affect vascular physiology and, together with platelet MVs, contribute to decreased CFR values. This hypothesis is summarized in Fig. 10. We also observed a positive correlation between platelet and endothelial MV concentrations. This is well in line with the report by Nomura et al. who observed a similar relationship for these types of MVs and soluble E-selectin in hypertensive patients (35).

A limitation of this study is the use of different cutoff CFR values, compared with the original study (25). We applied median CFR value for dividing patients into equally sized high and low CFR groups. However, similar results were obtained when using 2.5 as a CFR cutoff that usually defines low CFR (Supplemental Fig. S8). Also, due to technical limitations we were unable to quantify total number of MVs and thus MV concentration is reported.

We then investigated if the relationship between CFR and MVs is also reflected by their content. The proteomic analysis indicated that seven proteins were significantly increased in EVs isolated from the low CFR group and negatively correlating with CFR: BAFF, NEMO, resistin, AXL, perlecan, IGFBP7, and CD163. The general kinship of these proteins is that they all can be associated with a dysfunctional cardiovascular functionality. The proteomic evaluation was performed on total EVs and not platelet- or endothelial-derived subfractions that is a limitation of this analysis.

We chose to further examine BAFF, a TNF family molecule of predominantly myeloid origin. The cytokine stimulates B cell differentiation and promotes its survival (30). In a cardiovascular context, application of anti-BAFF-R antibody reduces the lesions (36) and depletion of B cells reduces progression of atherosclerosis in mice (37). Zouggari et al. (38) showed that circulating levels of BAFF correlate with adverse effects in patients with MI. Natorska et al. (29) speculate that B cell and BAFF-expressing macrophage accumulation contributes to increased inflammation and thrombosis, leading to vessel calcification and further progression of the disease. It is therefore interesting that patients with low CFR values express higher amounts of BAFF in plasma MVs.

Although leukocytes are considered the main source of BAFF (30), our analysis demonstrated the main source of circulating BAFF+ MVs to be platelets; thus, BAFF+ MVs represent a fraction of platelet MVs. This is supported by the results of Momoi et al. (39) and Finkielsztein et al. (40) who identified BAFF on platelets and megakaryocytes, respectively. The notion why BAFF is not cleaved by serine protease furin into the soluble form (41) can be explained by the presence of the serpin proteinase inhibitor 8 (PI8) in platelets (42). As PI8 is a furin inhibitor, it decreases BAFF shedding from platelets and platelet MVs, thus increasing the levels of circulating BAFF+ MVs.

To investigate whether proinflammatory conditions increase a release of BAFF+ platelet MVs, we stimulated a megakaryocyte cell line with TNFα and found that TNFα stimulation increased the production of BAFF-expressing pseudoplatelets. Thus, an inflammatory increase in circulating BAFF+ platelets subsequently resulting in increased levels of BAFF+ MVs could very well explain the higher levels of BAFF+ MVs that we found in the low CFR group. Since therapeutics has already been developed against BAFF, it could be an interesting target for developing drugs against microvascular dysfunction.

Most of the proteins that were negatively correlated with CFR have been mechanistically implicated in CVD. NF-κB activator NEMO causes transcriptional activation of inflammatory response genes (43, 44). Resistin is a cytokine that is mainly produced by immune cells and has connections with hypertension, diabetes, insulin resistance, and CVD (45). AXL is a tyrosine kinase receptor that has been tied to thrombosis, angiogenesis, MI, and ventricular modeling (46–48). We could speculate that AXL, via MVs, carries proatherogenic signals to ECs or platelets. Perlecan is a main constituent of extracellular matrix of the vessel wall. IGFBP7 is a component of endothelial Weibel–Palade bodies, to which it is specifically sorted via vWF binding (49). ECs release Weibel–Palade bodies via exocytosis and expose vWF upon, e.g., thrombin stimulation, thus during proatherogenic conditions. CD163 was another marker that positively correlated with CFR. CD163 protein and CD163+ macrophages have been detected in human atherosclerotic lesions (50, 51). Interestingly, it was also shown that concentration of circulating CD163 can predict CAD extent (50) and the soluble form of CD163 was proposed as a marker of macrophage engagement in inflammatory and proatherosclerotic processes (50). Increased levels of all these proatherogenic proteins could contribute to the development of endothelial dysfunction and low CFR.

The acoustic trapping method allows for isolation of not only vesicles of MV size, but also a wider spectrum of EVs. However, the majority of circulating MVs in plasma are derived from platelets (4, 5); thus, the results of the proteomic analysis may generally reflect the content of platelet MVs. It is also reflected by cellular origin of more than half of significantly different proteins—BAFF, IGFBP7, AXL, and NEMO have been identified in platelets, according to the PlateletWeb database. It is noteworthy that previous proteomic analyses of plasma MVs identified proteins of mainly platelet origin (17, 52, 53).

We further explored the proatherogenic effect of circulating MVs from the high and low CFR groups on ECs. ICAM-1 expression on ECs was assessed, as ICAM-1 is a marker of endothelial activation and plaque formation (54). Blood levels of ICAM-1 are elevated during progression of atherosclerosis (54–57). Our results showed that treatment of ECs with MVs from the low CFR group increased ICAM-1 expression, compared with the control cells. This supports our hypothesis that MVs circulating in plasma of patients with vascular dysfunction can play a role in amplifying proatherosclerotic status. Interestingly, ECs incubated with MVs from the high CFR group also exhibited a trend toward increased ICAM-1 expression, compared with the control cells. Patients from the high CFR group, despite more favorable CFR values, were diagnosed with CVD. Thus, circulating MVs in blood of these patients could also carry proatherogenic molecules, although to a lower extent than the low CFR group.

In summary, in the PROFLOW trial we explored the connection between CFR values and MVs in plasma from 220 patients with CAD. Our results confirm our initial hypothesis that the CFR magnitude is reflected in both levels of different MVs as well as in their content. We observed significantly increased platelet and endothelial MV levels in the low CFR group. Analysis of potential cardiovascular biomarkers in plasma EVs revealed a negative correlation between CFR and the levels of AXL, CD163, IGFBP7, NEMO, resistin, BAFF, and perlecan, respectively. As all the described proteins take part in inflammatory and proatherogenic processes, we propose that they collectively reflect the inferior vascular status of the low CFR group. It also suggests their potential clinical value as biomarkers of reduced blood flow and vascular status deterioration. Furthermore, humanized antibodies against, e.g., BAFF could be potentially tested as medications for cardiovascular disease.

## GRANTS

This work was supported by the Swedish Foundation for Strategic Research, the Knut and Alice Wallenberg Foundation, the Swedish Heart-Lung Foundation, the Swedish Research Council, and Region of Scania and ALF sources.

## DISCLOSURES

Elmir Omerovic and David Erlinge have received institutional grants from AstraZeneca. Li-Ming Gan is an employee of AstraZeneca. Thomas Laurell is a founder, board member, and shareholder of AcouSort AB, a university spin-off company that commercializes acoustofluidic technology. None of the other authors has any conflicts of interest, financial or otherwise, to disclose.

## AUTHOR CONTRIBUTIONS

P.B-G., E.O., and D.E., conceived and designed research; P.B-G., K.J., K.T., I.H., L-M.G., and S.S. performed experiments; P.B-G and K.J. analyzed data; P.B-G and K.J. interpreted results of experiments; P.B-G prepared figures; P.B-G and B.O. drafted manuscript; B.O., T.L., E.O., and D.E. edited and revised manuscript; P.B-G, K.J., K.T., I.H., L-M.G., S.S., B.O., T.L., E.O., and D.E. approved final version of manuscript.
